# Highlights of HRCT imaging in IPF

**DOI:** 10.1186/1465-9921-14-S1-S3

**Published:** 2013-04-16

**Authors:** N Sverzellati

**Affiliations:** 1 Scienze Radiologiche, Padiglione Barbieri, Azienda Ospealiero-Universitaria di Parma, V. Gramsci 14, 43100 Parma, Italy

**Keywords:** HRCT, Diagnosis, Honeycombing, Prognosis, UIP, Follow-up, IPF

## Abstract

High-resolution computed tomography (HRCT) imaging has a central role in the diagnosis of interstitial lung diseases, particularly in the evaluation of patients with suspected idiopathic pulmonary fibrosis (IPF). In approximately half of cases, HRCT scans are sufficient to allow a confident IPF diagnosis. Advances in HRCT scanning and interpretation have facilitated improved accuracy for use in diagnosing IPF, eliminating the need for a surgical biopsy in many patients. HRCT may also have a role to play in predicting the prognosis of the disease;. The role of routine follow-up with HRCT to monitor patients with IPF remains unclear due to lack of sufficient evidence, although, sometimes follow-up HRCT might be necessary to rule out progressive disease in patients with undetermined diagnosis. Advances in the field of HRCT imaging are discussed, along with insights into the clinical utility of this procedure in the diagnosis and management of IPF.

## Introduction

High-resolution computed tomography (HRCT) may substantially narrow the differential diagnosis for most cases with clinically suspected interstitial lung disease (ILD). Sometimes, HRCT may also provide a confident diagnosis without the need of the surgical biopsy. Furthermore, HRCT can quantify the extent of lung abnormalities and be used to make up composite indexes that better estimate disease severity and prognosis [1-3].

Indeed, HRCT is an essential tool for the evaluation of patients with suspected idiopathic pulmonary fibrosis (IPF) and has been increasingly used in several drug trials as a surrogate index for assessing treatment response [4-7]. The following highlights summarize the key technical requirements for improving HRCT imaging of patients with IPF, the HRCT role in IPF diagnosis and follow-up.

## Key technical requirements for best practice HRCT

The key technical requirements for ‘best practice’ HRCT are volumetric acquisition and thin-section reconstruction. Volumetric HRCT acquisition is now generally preferred to standard noncontiguous HRCT imaging (eg, 1mm scans with 10mm interval) because it improves both the identification of ancillary findings (e.g. lung nodules), and the characterization of patchy ILD [8]. Importantly, volumetric HRCT acquisition allows for better differentiation between honeycombing and traction bronchiectasis which may be proved crucial to diagnose or rule out IPF. Only volumetric imaging data provides multiplanar reformations (coronal and sagittal) of the entire lung improving the evaluation of abnormalities distribution and the extent of disease [9]. However, volumetric HRCT acquisition should only be performed if state-of-the-art multidetector-rows CT scanners (with at least 16 detector-rows) are available as the scanning time of early scanners (eg 4-6 detector-rows) is too long, with most patients unable to hold their breath long enough for a complete lung scan. . The major drawback of the volumetric technique is the high radiation dose exposure. Such a concern should be taken into account particularly when examining young patients with suspected ILD for whom standard noncontiguous HRCT should be considered as they deliver about one-fourth of the dose of standard volumetric HRCT [10]. Nevertheless, it is important to realize that reducing the tube current up to 20-30 mAs for volumetric HRCT scanning does not significantly impair the visual assessment of the lung parenchyma [11-14]. The increasing development of tools for reducing radiation exposure (e.g. automatic exposure control, iterative reconstruction) is reasonable assuming that diagnostic performance is not compromised [10,15].

Expiratory CT scanning might refine the differential diagnosis of fibrotic lung diseases by disclosing coexisting air trapping [16]. However it has yet to be proven whether or not the regular use of expiratory CT scanning provides any additional benefit in terms of diagnostic accuracy at the cost of increased radiation exposure.

HRCT scans are usually obtained with the patient in supine position. However, when limited (not extensive) ILD is suspected (by the clinical auscultation and/or the evaluation of the chest radiograph) prone imaging could be of use. Thus, the frequent finding of an amorphous increase in attenuation of the dependent lung (in the supine position, the postero-basal segments of the lower lobes) may mimic subtle interstitial abnormalities. However, this can be generally readily recognized as a normal finding if there are CT sections obtained in the prone position that confirm its reversibility [17].

## Definitive pattern of usual interstitial pneumonia (UIP) pattern at HRCT

Familiarity with the typical appearances of UIP on HRCT is important, as in the appropriate clinical setting, it is often sufficient for establishing a confident diagnosis of IPF without the need for surgical biopsy [6].

**Figure 1:**
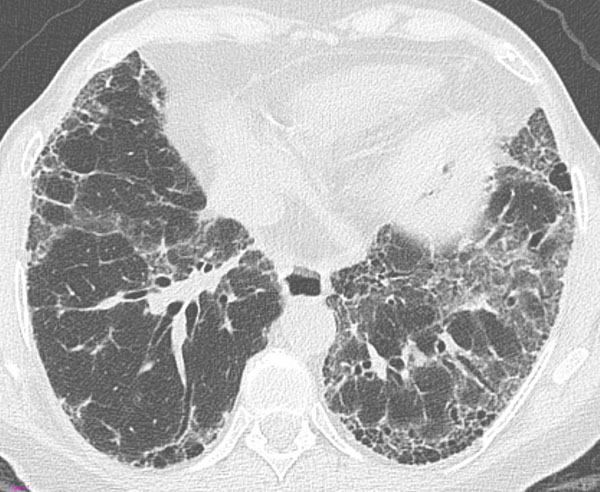
**Definite UIP pattern.** Transverse high-resolution computed tomography (HRCT) image shows subpleural basal honeycombing (more evident in the left lung) with traction bronchiectasis, reticular and ground-glass opacities.

The characteristic HRCT features of UIP are a reticular pattern with honeycombing, often associated with traction bronchiectasis; ground glass may be present, but is less extensive than reticular abnormality. Such abnormalities are characteristically basal and peripheral, though often patchy (Figure 1) [4].

Subpleural, basal honeycombing is indeed the most important, specific feature of the UIP pattern. On HRCT, honeycombing consists of clustered cystic airspaces, typically of comparable diameters of the order of 3–10 mm, though these may occasionally be as large as 2.5 cm. Honeycombing is characterized by well-defined walls [18]. In some cases of UIP with concurrent emphysema, emphysematous areas surrounded by ground-glass and/or reticular opacity may mimic honeycombing. In a study by Akira et al, it was shown that emphysematous changes complicated the interpretation of the CT findings thus leading to incorrect differential diagnosis between UIP and nonspecific interstitial pneumonia (NSIP) in more than half of the cases [19].

Although it's still not clear if IPF with coexisting emphysema represents a distinct clinical phenotype (combined pulmonary fibrosis and emphysema) or whether emphysema in these cases is simply a comorbidity, identifying coexisting emphysema may be important to better interpret pulmonary function test results [20-22]. Furthermore, it has been suggested that patients with IPF combined with emphysema are more likely develop pulmonary hypertension and then show a poorer outcome than those without emphysema [22,23].

HRCT features consistent with a definite UIP pattern may be asymmetrical in up to 25% of UIP cases. The right lung is most frequently involved. Such a pattern distribution of fibrosis is more common in UIP as compared with other fibrotic ILD [24].

However, a definite UIP-like pattern may be also seen in other disorders, such as fibrotic NSIP (especially when associated with emphysema), connective tissue diseases, chronic hypersensitivity pneumonitis, asbestosis, familial ILD, sarcoidosis, vasculitis and Hermansky Pudlak syndrome [16,25-28]. In such cases, the recognition of additional ancillary findings (e.g. pleural plaques, centrilobular nodules etc.) may help refine the differential diagnosis between an idiopathic and a known etiology-associated form (Table 1) [29].

**Table 1:** Ancillary CT findings suggesting other diagnoses in patients with a predominat UIP-like pattern.

Atypical CT features	Probable diagnosis
Centrilobular nodules Air trapping Relative sparing of bases	Hypersensitivity Pneumonitis

Pleural effusion; pleural thickening; esophageal dilation	Collagen Vascular disease

Pleural plaques	Asbestosis

Multiple septal or bronchovascular nodules in addition to reticulation	Fibrosing Sarcoid

## "Possible" and "inconsistent" HRCT patterns

**Figure 2:**
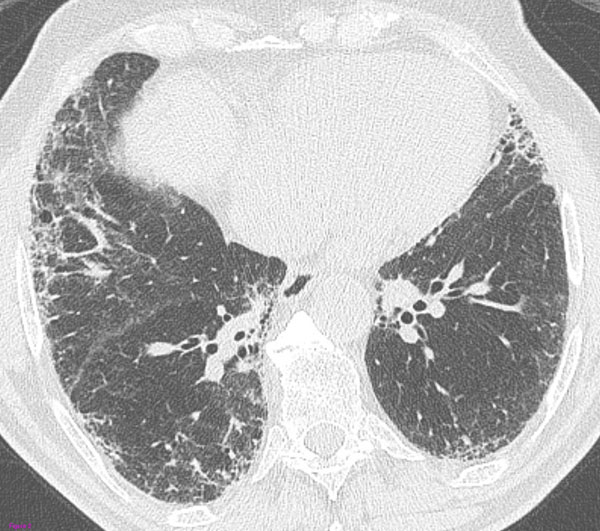
**Biopsy-proved UIP with a possible UIP pattern at HRCT.** The transverse HRCT image through the lower lobes shows patchy and peripheral reticular opacities without obvious honeycombing.

**Figure 3:**
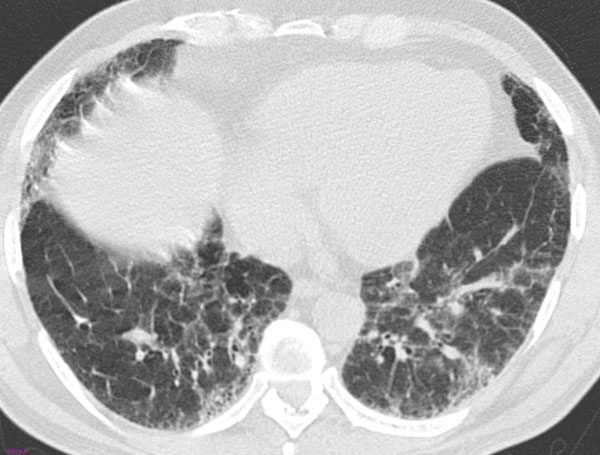
**Biopsy-proved UIP with an HRCT pattern inconsistent with UIP.** There are some patchy ground-glass and peripheral reticular opacities. which are more suggestive of nonspecific interstitial pneumonia (NSIP).

It is very important to realize that approximately half of cases with biopsy-proven UIP does not show a definite HRCT pattern owing to either the absence of typical findings (i.e. honeycombing) (Fig. 2), or the presence of findings (e.g. predominant ground-glass opacity, consolidation, nodules etc.) suggesting alternative diagnoses (Fig. 3) [3,30,31]. Such atypical HRCT patterns of UIP are very similar or identical to those of NSIP, although in clinical practice, they are likely to be misdiagnosed as a wider range of diseases (e.g. chronic hypersensitivity pneumonitis and sarcoidosis) [31].

In these patients, the absence of a typical HRCT pattern calls for surgical biopsy that reveals a diagnosis of UIP. The reason these patients fail to show the classic HRCT appearances of UIP is still unclear, although it thought that these are forms of initial disease or the expression of the coexistence of UIP and NSIP in different parts of the lung [10].

Thereby, the latest ATS/ERS/JRS/ALAT guidelines (2011) outlined the HRCT features that meet the criteria for "definite UIP", "possible UIP" and "inconsistent with UIP" patterns (Fig.1, 2, 3). Such classification system should be used and eventually combined with the histologic data to diagnose IPF [6].

Nevertheless, it has been shown that some HRCT indexes may predict both IPF diagnosis and prognosis. It seems that patients with IPF who have possible or inconsistent UIP by HRCT criteria have a shorter survival than those who show definite HRCT findings [30]. It has also been shown that amongst patients with a non definite UIP pattern, a higher HRCT score of the reticular abnormality and older age are predictive of a diagnosis of IPF [32].

## Role of follow-up HRCT

Serial HRCTs show increases in extent and fibrosis over months or years [25,33,34]. In drug trial setting HRCT is the main tool to assess in vivo the morphologic response to treatment [5,7]. However, the regular use of HRCT follow-up is still controversial in routine practice and not yet not recommended in clinically stable patients. Follow-up HRCT might be indicated in those patients presenting with an unexpected clinical-functional decline (i,e. to rule out acute exacerbations) or when there is a non definite UIP pattern on HRCT that cannot be characterized by lung biopsy.

Acute exacerbation should be suspected in patients with extensive or rapidly progressive new ground-glass opacity (but sometimes consolidation) occurring on a background of pre-existing fibrotic change typical of UIP/IPF HRCT [35,36]. In such cases, however.,HRCT interpretation should be carefully corroborated by clinical findings as acute exacerbations' HRCT features may overlap with those of other acute complications such as pulmonary infection and oedema.

## Summary

The role of HRCT imaging in the evaluation and diagnosis of patients with suspected IPF is central. When assessed by expert clinicians and radiologists, the presence of typical clinical features and definitive UIP findings on HRCT images are sufficient to allow a confident diagnosis of IPF. However, the spectrum of HRCT manifestations of UIP seems to be wider than commonly appreciated. HRCT has also been shown to have some prognostic value in predicting mortality although. further evidence is required to prove such a value in clinical practice. Routine HRCT follow-up is not currently recommended due to lack of sufficient evidence.

## Acknowledgements

The author thanks C. Trenam, I. Mandic and M. Smith of IntraMed Communications for editorial assistance in the preparation of the manuscript. Development of this article was supported by InterMune AG.

## Declarations

This article has been published as part of *Respiratory Research* Volume 14 Supplement 1, 2013:IPF in 2011 – Key updates on guidelines and therapeutics. The full contents of the supplement are available online at http://respiratory-research.com/supplements/14/S1. Publication of this supplement was supported by IntraMed Communications with funding from InterMune, AG. InterMune is the manufacturer of pirfenidone, a product mentioned in this article. The supplement originates from presentations given at the “AIR Event: Advancing IPF Research. Working together to translate IPF research into practice” held in Berlin in November 2011. The publication was proposed by IntraMed Communications and developed in consultation with the journal. All articles in the supplement have undergone the journal’s standard peer review process.

## References

[B1] Wells AU Desai SR Rubens MB Idiopathic pulmonary fibrosis: a composite physiologic index derived from disease extent observed by computed tomography Am J Respir Crit Care Med 2003 167 7 962 9 10.1164/rccm.2111053 12663338

[B2] Best AC Meng J Lynch AM Idiopathic pulmonary fibrosis: physiologic tests, quantitative CT indexes, and CT visual scores as predictors of mortality Radiology 2008 246 3 935 40 10.1148/radiol.2463062200 18235106

[B3] Sumikawa H Johkoh T Colby TV Computed tomography findings in pathological usual interstitial pneumonia: relationship to survival Am J Respir Crit Care Med 2008 177 4 433 9 10.1164/rccm.200611-1696OC 17975197

[B4] Lynch DA Godwin JD Safrin S High-resolution computed tomography in idiopathic pulmonary fibrosis: diagnosis and prognosis Am J Respir Crit Care Med 2005 172 4 488 93 10.1164/rccm.200412-1756OC 15894598

[B5] Demedts M Behr J Buhl R High-dose acetylcysteine in idiopathic pulmonary fibrosis N Engl J Med 2005 353 21 2229 42 10.1056/NEJMoa042976 16306520

[B6] Raghu G Collard HR Egan JJ An official ATS/ERS/JRS/ALAT statement: idiopathic pulmonary fibrosis: evidence-based guidelines for diagnosis and management Am J Respir Crit Care Med 2011 183 6 788 824 10.1164/rccm.2009-040GL 21471066 PMC5450933

[B7] Iwasawa T Ogura T Sakai F CT analysis of the effect of pirfenidone in patients with idiopathic pulmonary fibrosis Eur J Radiol 2012 22465123 10.1016/j.ejrad.2012.02.014

[B8] Mayo JR CT evaluation of diffuse infiltrative lung disease: dose considerations and optimal technique J Thorac Imaging 2009 24 4 252 9 10.1097/RTI.0b013e3181c227b2 19935222

[B9] Aziz ZA Padley SP Hansell DM CT techniques for imaging the lung: recommendations for multislice and single slice computed tomography Eur J Radiol 2004 52 2 119 36 10.1016/j.ejrad.2004.01.005 15489069

[B10] Bankier AA Tack D Dose reduction strategies for thoracic multidetector computed tomography: background, current issues, and recommendations J Thorac Imaging 2010 25 4 278 88 10.1097/RTI.0b013e3181eebc49 21042066

[B11] Naidich DP Marshall CH Gribbin C Arams RS McCauley DI Low-dose CT of the lungs: preliminary observations Radiology 1990 175 3 729 31 2343122 10.1148/radiology.175.3.2343122

[B12] Zwirewich CV Mayo JR Muller NL Low-dose high-resolution CT of lung parenchyma Radiology 1991 180 2 413 7 2068303 10.1148/radiology.180.2.2068303

[B13] Sverzellati N Zompatori M De Luca G Evaluation of quantitative CT indexes in idiopathic interstitial pneumonitis using a low-dose technique Eur J Radiol 2005 56 3 370 5 10.1016/j.ejrad.2005.05.012 15978764

[B14] Sverzellati N Guerci L Randi G Interstitial lung diseases in a lung cancer screening trial Eur Respir J 2011 38 2 392 400 10.1183/09031936.00201809 21233262

[B15] Baumueller S Winklehner A Karlo C Low-dose CT of the lung: potential value of iterative reconstructions Eur Radiol 2012 22 2597 2606 10.1007/s00330-012-2524-0 22699873

[B16] Silva CI Muller NL Lynch DA Chronic hypersensitivity pneumonitis: differentiation from idiopathic pulmonary fibrosis and nonspecific interstitial pneumonia by using thin-section CT Radiology 2008 246 1 288 97 18096541 10.1148/radiol.2453061881

[B17] Hansell DM Thin-section CT of the lungs: the Hinterland of normal Radiology 2010 256 3 695 711 10.1148/radiol.10092307 20720066

[B18] Hansell DM Bankier AA MacMahon H McLoud TC Muller NL Remy J Fleischner Society: glossary of terms for thoracic imaging Radiology 2008 246 3 697 722 10.1148/radiol.2462070712 18195376

[B19] Akira M Inoue Y Kitaichi M Yamamoto S Arai T Toyokawa K Usual interstitial pneumonia and nonspecific interstitial pneumonia with and without concurrent emphysema: thin-section CT findings Radiology 2009 251 1 271 9 10.1148/radiol.2511080917 19221055

[B20] Kurashima K Takayanagi N Tsuchiya N The effect of emphysema on lung function and survival in patients with idiopathic pulmonary fibrosis Respirology 2010 15 5 843 8 10.1111/j.1440-1843.2010.01778.x 20546187

[B21] Mura M Zompatori M Pacilli AM Fasano L Schiavina M Fabbri M The presence of emphysema further impairs physiologic function in patients with idiopathic pulmonary fibrosis Respir Care 2006 51 3 257 65 16533415

[B22] Cottin V Cordier JF Combined pulmonary fibrosis and emphysema: an experimental and clinically relevant phenotype Am J Respir Crit Care Med 2005 172 12 1605 author reply -6 10.1164/ajrccm.172.12.1605 16339012

[B23] Mejia M Carrillo G Rojas-Serrano J Idiopathic pulmonary fibrosis and emphysema: decreased survival associated with severe pulmonary arterial hypertension Chest 2009 136 1 10 5 10.1378/chest.08-2306 19225068

[B24] Tcherakian C Cottin V Brillet PY Progression of idiopathic pulmonary fibrosis: lessons from asymmetrical disease Thorax 2011 66 3 226 31 10.1136/thx.2010.137190 20880868

[B25] Silva CI Muller NL Hansell DM Lee KS Nicholson AG Wells AU Nonspecific interstitial pneumonia and idiopathic pulmonary fibrosis: changes in pattern and distribution of disease over time Radiology 2008 247 1 251 9 10.1148/radiol.2471070369 18270375

[B26] Song JW Do KH Kim MY Jang SJ Colby TV Kim DS Pathologic and radiologic differences between idiopathic and collagen vascular disease-related usual interstitial pneumonia Chest 2009 136 1 23 30 10.1378/chest.08-2572 19255290

[B27] Padley SP Padhani AR Nicholson A Hansell DM Pulmonary sarcoidosis mimicking cryptogenic fibrosing alveolitis on CT Clin Radiol 1996 51 11 807 10 10.1016/S0009-9260(96)80011-0 8937326

[B28] Copley SJ Wells AU Sivakumaran P Asbestosis and idiopathic pulmonary fibrosis: comparison of thin-section CT features Radiology 2003 229 3 731 6 10.1148/radiol.2293020668 14576443

[B29] Sverzellati N De Filippo M Bartalena T Piciucchi S Zompatori M High-resolution computed tomography in the diagnosis and follow-up of idiopathic pulmonary fibrosis Radiol Med 2010 115 4 526 38 10.1007/s11547-010-0512-5 20082223

[B30] Flaherty KR Thwaite EL Kazerooni EA Radiological versus histological diagnosis in UIP and NSIP: survival implications Thorax 2003 58 2 143 8 10.1136/thorax.58.2.143 12554898 PMC1746568

[B31] Sverzellati N Wells AU Tomassetti S Biopsy-proved idiopathic pulmonary fibrosis: spectrum of nondiagnostic thin-section CT diagnoses Radiology 2010 254 3 957 64 10.1148/radiol.0990898 20177106

[B32] Fell CD Martinez FJ Liu LX Clinical predictors of a diagnosis of idiopathic pulmonary fibrosis Am J Respir Crit Care Med 2010 181 8 832 7 10.1164/rccm.200906-0959OC 20056903 PMC2854332

[B33] Akira M Sakatani M Ueda E Idiopathic pulmonary fibrosis: progression of honeycombing at thin-section CT Radiology 1993 189 3 687 91 8080483 10.1148/radiology.189.3.8080483

[B34] Jeong YJ Lee KS Muller NL Usual interstitial pneumonia and non-specific interstitial pneumonia: serial thin-section CT findings correlated with pulmonary function Korean J Radiol 2005 6 3 143 52 10.3348/kjr.2005.6.3.143 16145289 PMC2685037

[B35] Lloyd CR Walsh SL Hansell DM High-resolution CT of complications of idiopathic fibrotic lung disease Br J Radiol 2011 84 1003 581 92 10.1259/bjr/65090500 21697412 PMC3473493

[B36] Akira M Kozuka T Yamamoto S Sakatani M Computed tomography findings in acute exacerbation of idiopathic pulmonary fibrosis Am J Respir Crit Care Med 2008 178 4 372 8 10.1164/rccm.200709-1365OC 18451320

